# Antiviral responses in peripheral and brain neurons

**DOI:** 10.1016/j.coi.2025.102678

**Published:** 2025-10-14

**Authors:** Brian Imbiakha, Hana Janova, Michael S Diamond

**Affiliations:** 1 Department of Medicine, Washington University School of Medicine, Saint Louis, MO 63110, USA; 2 Department of Pathology and Immunology, Washington University School of Medicine, Saint Louis, MO 63110, USA; 3 Department of Molecular Microbiology, Washington University School of Medicine, Saint Louis, MO 63110, USA; 4 The Andrew M. and Jane M. Bursky Center for Human Immunology and Immunotherapy Programs, Washington University School of Medicine, Saint Louis, MO 63110, USA

## Abstract

Neurotropic viruses represent a public health challenge due to their ability to cause severe neurological conditions, including meningitis, encephalitis, and paralysis. Although many studies have investigated the immune responses to viral infections in the brain and other nervous system targets, most have focused on the effects of resident cells, such as microglia and astrocytes, and infiltrating immune cells, including neutrophils, monocytes, and T cells. However, emerging evidence has demonstrated that neurons themselves mount antiviral responses that suppress viral replication directly and enhance the inhibitory functions of adjacent glial and infiltrating immune cells. In this review, we discuss the intrinsic and extrinsic antiviral responses of neurons, highlighting mechanisms by which they detect viruses and initiate inhibitory responses to protect the nervous system from viral invasion and injury.

Cell-intrinsic antiviral immune responses serve as a primary defense mechanism to limit the spread of infection by interfering with many steps of the viral life-cycle, including entry, translation, replication, assembly, and virion morphogenesis. Most cells, including neurons, detect viral infections by recognizing their nucleic acids (RNA or DNA) in the endosome, cytoplasm, or nucleus as non-self, pathogen-associated molecular patterns. This detection is mediated by well-characterized pattern recognition receptors (PRRs), including Toll-like receptors (TLRs), retinoic acid-inducible gene-I-like receptors (RIG-I), protein kinase R (PKR), and cytosolic and nuclear DNA sensors (Figure 1) [1–7]. PRR engagement and subsequent signaling cascades promote expression of antiviral genes and proinflammatory cytokines, including type I interferons (IFNs), which have a central role in orchestrating inhibitory responses in cells. IFNs activate antiviral defenses in a paracrine and/or autocrine manner through Janus-activated kinase (JAK) and signal transducer and activator of transcription (STAT) signaling pathways that induce hundreds of IFN-stimulated genes (ISGs) in a given cell type [8]. Several ISGs have demonstrated antiviral effector roles in neurons, including members of the IFN-induced protein with tetratricopeptide repeats (IFIT) family, the IFN-induced transmembrane protein family (IFITM), RSAD2, IRG1, and IFI27 [9–12]. Although IFN-dependent immune responses contribute to antiviral activity in the peripheral nervous system (PNS) and central nervous system (CNS), other cell-intrinsic inhibitory mechanisms, including host restriction factors such as the transmembrane protein with EGF-like and two follistatin-like domains 1(TMEFF1), microRNA (miR-138), and autophagic responses, have been described in neurons (Table 1) [13–15]. Below, we discuss the strategies that neurons of the PNS and CNS use to limit viral infection and spread.

## Antiviral defenses of neurons in the peripheral nervous system

Many neuroinvasive viruses initially infect peripheral tissues, often in the skin or at mucosal surfaces. If viruses are not cleared rapidly by local immune responses, they can gain access to the PNS and CNS. In this section, we focus on antiviral immunity of trigeminal, dorsal root ganglion (DRG), and olfactory sensory neurons, since their infection can serve as a gateway for virus entry into the CNS.

### Trigeminal and dorsal root ganglionic sensory neurons

Herpesviruses, especially herpes simplex virus 1 (HSV-1), infect peripheral neurons and spread to the DRG and associated neuronal cells. HSV-1 initially replicates in epithelial cells in the skin or genital mucosa before infecting sensory neurons through axonal terminals. After retrograde spread to neuronal cell bodies, HSV-1 establishes a latency stage that can persist for decades [16], whereby the HSV genome remains transcriptionally silent except for expression of latency-associated transcripts (LATs). DRG neurons can restrain HSV-1 spreading to CNS by promoting latency through expression of a microRNA (miR-138) that represses expression of ICP0, a viral transactivator of lytic gene expression [15]. HSV-1 LATs can prevent DRG neuronal death, which facilitates viral persistence. [17]. Despite limited viral protein production in DRG neurons, LATs generate peptides that are presented on major histocompatibility complex class I molecules, enabling CD8^+^ T cells to suppress HSV-1 reactivation in neurons through noncytolytic mechanisms [18]. Once reactivation occurs, due to stress, immunosuppression, ultraviolet light irradiation, or hormonal changes, HSV-1 transits in an anterograde direction along sensory nerves to peripheral epithelial cells, resulting in herpetic lesions. DRG neurons produce little type I IFN in response to HSV-1 infection [13], and even if produced (or exogenously added), the response fails to efficiently block HSV replication or trigger cell death [13,19,20].

Instead, restriction of HSV infection in DRG neurons is principally achieved via autophagy [13,21]. As part of this process, viruses that enter neurons upon membrane fusion or endocytosis are sequestered within double-membrane-bound vesicles and targeted to autolysosomes for degradation [22]. Notwithstanding this defense mechanism, HSV-1 encodes an immune evasion protein, ICP34.5, which binds to the autophagy protein Beclin 1 in neurons and inhibits autophagy. HSV strains that are engineered with deletions in *ICP34.5* genes are attenuated in immunocompetent animals, fail to spread to the brain [21], have been considered for vaccine development [23], and have also shown promise as a directly administered treatment for brain tumors, such as glioblastoma [24].

### Olfactory sensory neurons

The dendrites of bipolar olfactory sensory neurons extend to the epithelial surface of the nasal passages, where they detect odors. Their axons terminate in the olfactory bulb of the brain, making them a potential conduit for pathogens into the CNS [25]. Possibly because of this, olfactory sensory neurons have evolved strategies to contain the spread of viruses. One defense is apoptosis, as shown for rhabdoviruses and influenza A virus [26,27]. Nasal delivery of rhabdoviruses induces apoptosis in crypt olfactory sensory neurons via interaction of the viral G glycoprotein with the signaling receptor tropomyosin-related kinase A (TrkA); this interaction triggers electrical activation of neurons and programmed cell death [27]. As a second antiviral defense, olfactory neurons use the type I IFN signaling response. Influenza B virus infection of olfactory neurons is cleared by expression of ISGs, including IFIT1, IFIT3, RSAD2, and OAS [10]. Infected olfactory sensory neurons also can trigger production of type I IFNs and expression of ISGs in uninfected brain regions at long distances, possibly through IFN transit via the cerebrospinal fluid, as described after vesicular stomatitis virus (VSV) and cytomegalovirus (CMV) infections [28].

## Antiviral defenses of neurons in the brain

Given that viruses can kill neurons, which have limited regenerative capacity, they pose a threat to the function of the brain and other components of the CNS. Consequently, the brain has evolved physical barriers and mechanisms that restrict neuroinvasion [29,30]. However, if viruses breach these defenses, neurons and other cells can detect their invasion and mount antiviral responses that limit replication while preserving cell viability and function. Defects in intrinsic neuronal antiviral responses linked to inborn errors of the RNA sensor TLR3, the RNA lariat debranching enzyme DBR1, and the RNA processing protein SNORA31 and their association with viral encephalitis have been reviewed elsewhere [31]. In this section, we describe how neurons in the brain sense viral infection and induce inhibitory type I IFN-dependent and independent responses, and how viruses counteract these intrinsic host defenses.

### Virus detection and induction of interferon-dependent and independent antiviral responses

**Figure 2:**
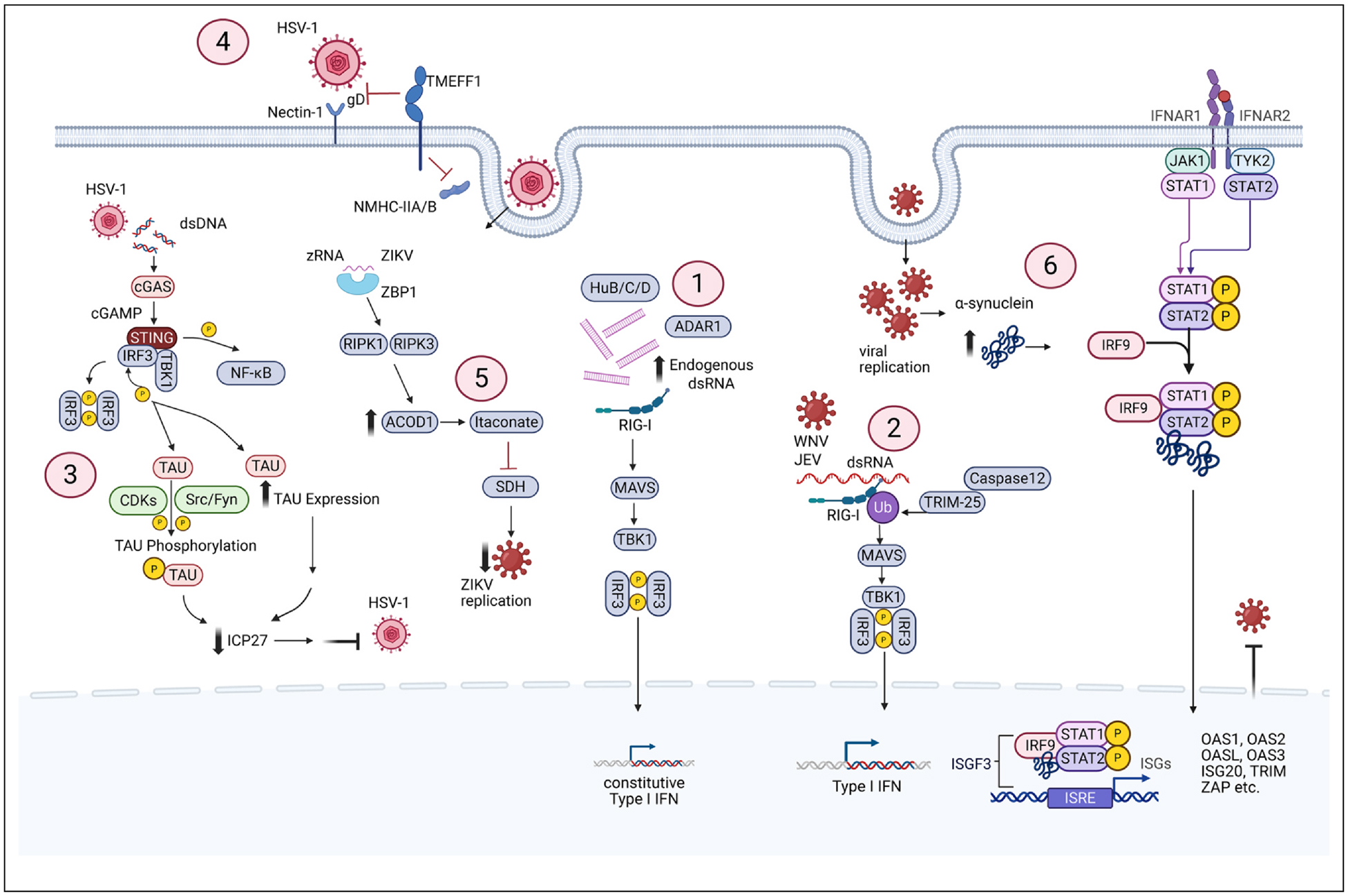
Alternative antiviral sensing and signaling pathways in neurons. **(1)** Double-stranded host RNA is enriched in neurons by the ELAVL family of proteins (HuB/C/D) and is further modified by ADAR1, resulting in the constitutive expression of low levels of type I IFN. **(2)** Upon sensing WNV RNA, RIG-I is activated through caspase-12-driven TRIM25-mediated ubiquitination, leading to expression of type I IFNs. **(3)**. A noncanonical cGAS–STING signaling pathway contributes to TAU phosphorylation, which reduces expression of HSV-1 ICP27, a protein required for HSV-1 replication. **(4)** HSV-1 entry into neurons can be inhibited by the neuron-specific antiviral factor TMEFF1, which blocks HSV-1 gD binding to Nectin-1 and gB binding to NMHC-IIA/B. **(5)** ZBP1 detects ZIKV Z-RNA, activating RIPK1 and RIPK3 and upregulating ACOD1. ACOD1 induces itaconate production, which inhibits SDH, an enzyme that generates metabolites necessary for ZIKV replication. **(6)** Type I IFN signaling promotes colocalization of α-synuclein with STAT-2, enhancing ISG induction.

Much of our knowledge on viral responses in neuronal cells of the brain comes from studies with purified neurons isolated from the cerebral cortex. Cortical neurons, which reside in outer layers of the brain, often encounter viruses that enter the brain parenchyma from the meninges, as demonstrated after lymphocytic choriomeningitis virus infection [32]. Cortical neurons constitutively express several PRRs, including TLR3, melanoma differentiation-associated gene 5 (MDA5), RIG-I, and PKR [1,6]. A unique feature of cortical neurons is that they also basally express low levels of type I IFN under homeostatic conditions (Figure 2) [1,6], which may enable early control of some viruses, including HSV-1, Zika virus (ZIKV), and Sindbis virus (SINV) [1,6]. Basal expression of type I IFNs in cortical neurons also has been attributed to sensing of host dsRNA that is enriched by the embryonic lethal, abnormal vision-like (ELAVL) family of genes (HuB, HuC, and HuD) and modified by the dsRNA-editing enzyme adenosine deaminase acting on RNA 1 (ADAR1) [1].

Neuronally expressed RIG-I in the cytoplasm recognizes viral RNA and promotes antiviral and IFN gene induction [4]. Silencing of RIG-I in primary murine cortical neurons results in increased susceptibility to infection by two related orthoflaviviruses, Japanese encephalitis (JEV) [4] and West Nile (WNV) viruses [33], as well as decreased expression of the pro-inflammatory cytokines interleukin 12, tumor necrosis factor (TNF), and IL-6. Caspase-12 regulates RIG-I activation and the ensuing type I IFN induction in mouse cortical neurons by promoting tripartite motif-containing 25 (TRIM-25) mediated ubiquitination of RIG-I, an activating post-translational modification that facilitates engagement with the downstream signaling adaptor molecule mitochondrial antiviral-signaling protein [34]. *Casp12*^−/−^ mice exhibited higher WNV burden in the brain and increased mortality [33], although the phenotypes *in vivo* were not linked to differences in RIG-I activity.

Cyclic GMP-AMP synthase (cGAS) is a cytosolic DNA sensor that generates cGAMP upon binding of viral DNA. cGAMP binds to the adaptor molecule stimulator of interferon genes (STING), resulting in the induction of inflammatory mediators, including IL-6, TNF, and type I IFNs. This pathway has been shown to contribute to intrinsic antiviral defenses in neurons of the entorhinal cortex [35]. In primary neuron cultures, HSV-1 infection activates a non-canonical cGAS-STING signaling cascade (via TBK1, SRC, and FYN) that promotes phosphorylation of the microtubule-associated protein TAU, which downregulates expression of ICP27, a viral protein essential for HSV-1 replication, and enhances neuronal survival [35]. As HSV-1 reactivation correlates with a higher incidence of Alzheimer’s disease [36], it has been suggested that antiviral mechanisms that preserve neuronal viability (e.g. through TAU phosphorylation) might come at the cost of promoting neurodegeneration. Whether antiviral-induced TAU protein contributes to neurodegeneration or if Tauopathies limit viral replication requires further investigation.

TMEFF1 was recently identified as an IFN-independent, cell surface-expressed, neuronal cell-specific restriction factor against HSV-1 [14,37]. TMEFF1 is highly expressed in neurons of the cerebral cortex, hypothalamus, brainstem, olfactory bulb, and spinal cord. HSV-1 infection in *Tmeff1*^−/−^ mice is elevated in the brainstem compared to infected wild-type mice [14]. The extracellular domain of TMEFF1 interacts with the HSV-1 gD-binding site on Nectin-1, whereas its intracellular domain interacts with the HSV-1 gB binding site on non-muscle myosin heavy chains IIA and IIB (NMHC-IIA/B); both binding activities enable TMEFF1 to block HSV entry [14,37]. These findings open avenues for the development of CNS-specific therapeutics against HSV using soluble and headless TMEFF1 decoy receptors targeting nectin-1 and non-muscle myosin heavy chains.

In many brain neurons, type I IFN responses have key antiviral effects. Experiments in primary cortical and cerebellar neurons established that receptor-interacting protein kinase-3 (RIPK3) is a key inducer of type I IFNs and ISGs during WNV and Langat virus (LGTV) infection, while also limiting necroptosis [38,39]. Apart from this, RIPK3 restricts infection by ZIKV, another orthoflavivirus, through a distinct mechanism in primary cortical neurons [40]. Z-conformation nucleic acid binding protein 1 (ZBP1) senses ZIKV RNA and activates both RIPK1 and RIPK3, leading to increased expression of aconitase decarboxylase (ACOD1), a mitochondrial enzyme. ACOD1 alters neuronal metabolism by producing itaconate, which inhibits succinate dehydrogenase (SDH), an enzyme that provides energy and cellular metabolites that likely are needed for ZIKV replication [40]. However, a direct linkage of the specific downstream metabolites induced by SDH and their roles in ZIKV replication has not been defined.

In studies with VSV in mice, type I IFN signaling in both neurons and astrocytes restricts viral spread from the olfactory bulb to other brain regions by recruiting and activating microglia [41]. Additionally, type I IFN signaling restricts infection of neurotropic orthoflaviviruses and alphaviruses (WNV and Venezuelan equine encephalitis virus (VEEV), respectively) in mouse brains and human dopaminergic neurons through α-synuclein-dependent mechanisms [42,43]. α-synuclein colocalizes with phosphorylated STAT2 in dopaminergic neurons, and α-synuclein-deficient mice exhibited decreased expression of several ISGs following WNV infection [43]. α-synuclein expression also restricts WNV and VEEV infection in cortical and striatal neurons by modulating cell death through caspase-3 expression [42].

## MicroRNA-mediated effects on neuronal antiviral responses

MicroRNAs (miRs) are conserved, non-coding RNAs that regulate expression of specific mRNAs. Several miRs act as antagonists of neuronal antiviral responses and accordingly are induced by viruses as a mechanism of host defense evasion. JEV infection upregulated miR-301a expression in a hippocampal neuron cell line and primary neuron cultures, which resulted in suppression of IFN-β expression and increased viral replication and cytolysis [44]. MiR-301a exerts its effect by downregulating IFN regulatory factor 1 and the signaling protein suppressor of cytokine signaling 5 [44]. In other studies, miR-132 has been implicated as a proviral factor in WNV infection [9]. MiR-132 levels are higher in more permissive cortical neurons than cerebellar granule neurons, and repress key antiviral and ISGs, including STAT1, ISG15, and IFI44 [9].

## Concluding remarks

Neuronal antiviral responses contribute to restricting viral infections and controlling inflammation, which can adversely affect the function and survival of cells with limited capacity for regeneration. Depending on the type of neuron, its location in the body, and the specific pathogen involved, neurons have developed unique intrinsic and extrinsic antiviral mechanisms. To prevent viral invasion of the brain, infected olfactory neurons can induce ISGs in posterior regions of the brain, or they can sacrifice themselves by activating apoptosis programs [27]. Sensory neurons of the DRG can use autophagic pathways to limit viral infection and spread to the brain and CNS [13,21]. Host defense responses by neurons in the brain have been detailed in multiple studies and highlighted by what happens with inborn errors of immunity, particularly TLR3 deficiencies and their linkage to herpesvirus encephalitis [6,45,46]. Discoveries such as the neuron-specific IFN-independent restriction factor TMEFF1, non-canonical RIPK3 activation, and constitutive type I IFN production highlight additional antiviral defense programs, some of which are unique to neurons [1,14,37–40]. While basal levels of type I IFN in neurons likely contribute to antiviral responses, they also may modulate other functions in the CNS, such as microglia activation and triggering phagocytosis of myelin debris [47].

Although *in vitro* infection models have revealed neuronal defense responses, most studies use cells from the cerebral cortex or cerebellum or from limited sources of peripheral neurons. Evidence that neurons from different brain regions respond variably to viral infection [9] in combination with the differential tropism of viruses for different brain regions [48,49] suggests that we may have a biased and incomplete view of neuronal immunity, with a need to examine a greater number of subtypes for host–pathogen interactions. The field might benefit from the use of neuronal organoids or organotypic slice cultures that combine different neuronal lineages in the context of other resident cells (e.g. microglia, astrocytes, oligodendrocytes, pericytes, or endothelial cells) to clarify effects on neuronal immunity. Complementary *in vivo* approaches, including neuron-targeted adeno-associated viruses [50] (carrying Cre recombinases), single-cell or nuclear RNA sequencing of neuronal subgroups, and surgical techniques paired with optogenetics, could provide a more granular analysis of how specific neuronal populations and brain regions respond to different viral infections. Such tools and approaches might help determine how modulation of neuronal antiviral responses shapes resistance to neurotropic viruses and inform strategies for developing antiviral molecules with activity for neurons of the PNS and CNS.

## Figures and Tables

**Figure 1:**
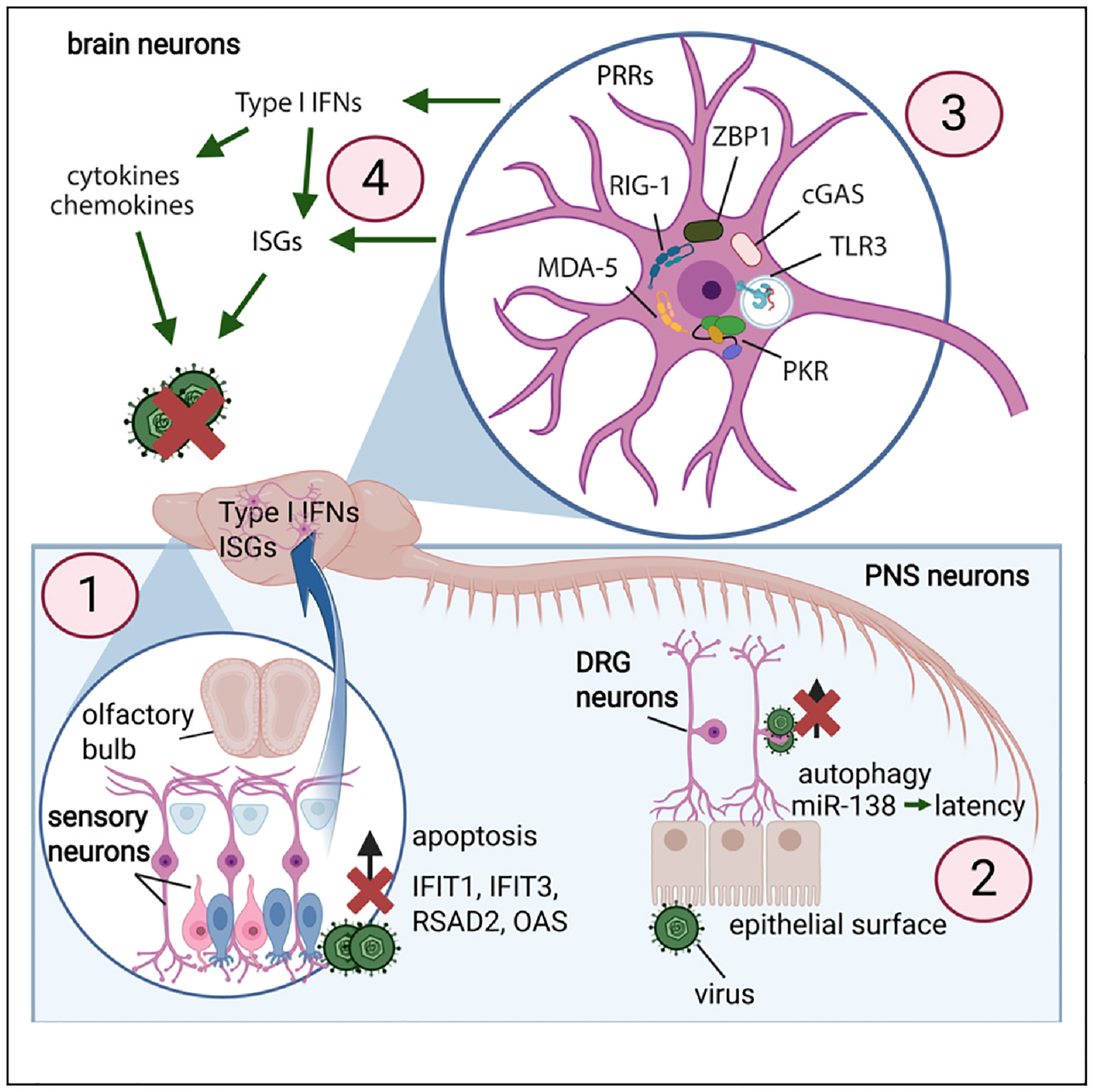
Major PRRs and antiviral mechanisms of PNS and CNS neurons. **(1)** Olfactory sensory neurons, located in the olfactory epithelium of the nasal cavity, limit viral spread through induction of apoptosis, expression of ISGs such as IFIT1, IFIT3, RSAD2, and OAS, and by initiating type I IFN production that induces ISG expression in uninfected brain regions. **(2)** Infected DRG neurons, positioned between the dorsal horn of the spinal cord and peripheral nerve terminals, primarily inhibit viral infections through autophagy or expression of microRNA miR-138, which promotes viral latency. **(3)** To detect RNA viruses, neurons utilize PRRs including MDA5, RIG-I, PKR, TLR3, and ZBP1. DNA viruses are recognized by PRRs, including cGAS. **(4)** Upon sensing viral RNA or DNA, these receptors activate signaling cascades that lead to ISG expression and type I IFN production, which can directly inhibit multiple steps in the virus lifecycle or stimulate the release of cytokines and chemokines to recruit and modulate infiltrating immune cells.

**Table 1:** Neuronal antiviral mechanisms.

Virus	Neuron type/brain region	Defense mechanisms	Antiviral outcome
HSV-1	Dorsal root ganglions (DRG)	- MiR-138 induced latency [15].	- HSV-1 latency, neuronal survival, and decreased mortality.
	Cortical Neurons	- Host dsRNA induced basal type I IFN [1]. - TLR3, IFN-β [6]. - TMEFF1 mediated blockade of viral entry [14,37].	- Neuronal protection from virus infections. - Constitutive basal type I IFN expression limits virus replication and cell death. - Inhibits HSV-1 interaction with Nectin-1 and non-muscle heavy chains, impairing virus entry.
	Entorhinal cortex	- Non-canonical cGAS-STING activation and TAU phosphorylation via TBK1, SRC, and FYN [35].	- Decreased ICP27 expression, reduced virus replication, and increased neuronal viability.
Rhabdoviruses	Olfactory sensory neurons	- TrKA induced apoptosis [27]	- Increased pro-inflammatory responses in olfactory tissues and decreased virus replication.
Influenza B	Olfactory sensory neurons	- Rapid ISG induction (IFIT1, IFIT3, PKR, RSAD2, OAS1A, ISG15) [10].	- Decreased influenza B virus replication.
ZIKV	Cortical Neurons	- Host dsRNA induced basal type I IFN [1]. - ZBP1 mediated activation of RIPK1 and RIPK3 and modulation of neuronal metabolism [40].	- Neuronal protection from virus infections. - Altered neuronal metabolism and suppression of ZIKV replication.
SINV	Cortical Neurons	- Host dsRNA induced basal type I IFN [1].	- Neuronal protection from virus infections.
JEV	Cortical Neurons	- RIG-I [4].	- Increased cytokine and chemokine production and decreased JEV replication.
WNV	Cortical Neurons	- RIG-I and caspase 12 [33]. - α-synuclein mediated caspase-3 activation [42].	- Type I IFN modulation via TRIM-25 mediated ubiquitination of RIG-I. - Decreased WNV replication in the CNS.
	Cortical and cerebellar granule cell neurons	- RIPK3 mediated type I IFN and ISG induction [38].	- Restriction of WNV replication.
VEEV	Striatal Neurons	- α-synuclein mediated caspase-3 activation [42].	- Decreased VEEV replication in the CNS.
LGTV	cerebellar granule cell neurons	- RIPK3 mediated type I IFN and ISG induction [39].	- Restriction of LGTV replication and decreased pathology.
VSV, CMV	Olfactory sensory neurons	- Activation of IFN-α/β responses and ISGs expression in the uninfected brain regions [28].	- Decreased VSV replication and spread beyond the olfactory bulb.
